# New Diabetes Therapies and Diabetic Kidney Disease Progression: the Role of SGLT-2 Inhibitors

**DOI:** 10.1007/s11892-018-0992-6

**Published:** 2018-03-27

**Authors:** Claire C. J. Dekkers, Ron T. Gansevoort, Hiddo J. L. Heerspink

**Affiliations:** 1 Department of Clinical Pharmacy and Pharmacology, University of Groningen, University Medical Center Groningen, De Brug 50D-1-015; EB70 University Medical Center Groningen, P.O. Box 30001, 9700 AD Groningen, the Netherlands; 2 Division Nephrology, Department of Internal Medicine, University of Groningen, University Medical Center Groningen, PO 30.001, 9700 RB, Groningen, the Netherlands

**Keywords:** Sodium-glucose co-trasporter-2 inhibitor, Type 2 diabetes, Chronic kidney disease, Pharmacology, Clinical trials

## Abstract

**Purpose of Review:**

Sodium-glucose co-transporter 2 (SGLT-2) inhibitors have emerged as a promising drug class for the treatment of diabetic kidney disease. Developed originally as glucose-lowering drugs by enhancing urinary glucose excretion, these drugs also lower many other renal and cardiovascular risk factors such as body weight, blood pressure, albuminuria, and uric acid. Results from the EMPA-REG OUTCOME and CANVAS trials show that these salutary effects translate into a reduction in cardiovascular outcomes and have the potential to delay the progression of kidney function decline. This review summarizes recent studies on the mechanisms and rationale of renoprotective effects.

**Recent Findings:**

Effects of SGLT-2 inhibitors on the kidney are likely explained by multiple pathways. SGLT-2 inhibitors may improve renal oxygenation and intra-renal inflammation thereby slowing the progression of kidney function decline. Additionally, SGLT-2 inhibitors are associated with a reduction in glomerular hyperfiltration, an effect which is mediated through increased natriuresis and tubuloglomerular feedback and independent of glycemic control. Analogous to diabetic kidney disease, various etiologies of non-diabetic kidney disease are also characterized by single nephron hyperfiltration and elevated albuminuria. This offers the opportunity to reposition SGLT-2 inhibitors from diabetic to non-diabetic kidney disease. Clinical trials are currently ongoing to characterize the efficacy and safety of SGLT-2 inhibitors in patients with diabetic and non-diabetic kidney disease.

**Summary:**

The glucose-independent hemodynamic mechanisms of SGLT-2 inhibitors provide the possibility to extend the use of SGLT-2 inhibitors to non-diabetic kidney disease. Ongoing dedicated trials have the potential to change clinical practice and outlook of high-risk patients with diabetic (and non-diabetic) kidney disease.

## Introduction

The worldwide prevalence of diabetes mellitus will continue to increase in the next decades from 415 million people in 2015 to 642 million in 2040 [1]. Approximately 40% of all patients with diabetes will develop diabetic kidney disease (DKD), and a substantial number of these patients will progress to end-stage renal disease [2]. Diabetic kidney disease is also independently associated with increased risk of cardiovascular disease and a significant reduction in life expectancy [2, 3]. Consequently, it places a heavy burden on individual patients and on national health budgets. Recent studies indicate that the 10-year mortality rates of patients with DKD equal average mortality rates of all cancers [4, 5]. There is thus a strong rationale to develop new interventions to slow the progression of DKD.

Current treatments to prevent or delay kidney (as well as cardiovascular) complications in patients with diabetes focus on lowering blood pressure, HbA1c, body weight, albuminuria, and cholesterol. Targeting these multiple risk factors reduce the risk of cardiovascular disease and kidney function decline [6, 7]. Nevertheless, many patients do not reach their target blood pressure, blood glucose levels, and/or lipid levels.

Recently, several strategies have been tested to improve the prognosis of patients with diabetes. One of these strategies was to examine the effects of intensive compared with conventional glucose control on cardiovascular complications. Several large clinical trials in patients with type 2 diabetes showed that aggressive glucose lowering did not result in a reduced risk for macrovascular complications [8, 9]. The ACCORD trial even showed that intensive glucose lowering increased mortality rates compared with conventional glucose control [10]. These findings, in combination with initial concerns about the safety of rosiglitazone, led the FDA to mandate that the cardiovascular safety of all new glucose-lowering drugs must be investigated in post-marketing clinical outcome trials. As a result, many large cardiovascular outcome trials have been completed the last few years or are ongoing. These trials are designed to demonstrate cardiovascular safety and are powered to show non-inferiority compared with control treatment. They have provided important insight in the efficacy and safety of various glucose-lowering drug classes which would likely have been unknown if the FDA mandate had not been in place. The first cardiovascular outcome trials tested the effects of dipeptidyl-peptidase-4 (DDP-4) inhibitors and demonstrated that these agents have largely neutral effects on cardiovascular and renal outcomes [11–13]. Glucagon-like-peptide-1 receptor agonist (GLP-1 RA) appeared to have a favorable cardiovascular safety profile and two of them, liraglutide and semaglutide, reduce both cardiovascular risk and albuminuria progression [14–17]. All these trials enrolled patients at high cardiovascular risk. Whether these agents slow progression of kidney function decline could not be appropriately established since on average the enrolled population was at low risk of kidney function loss. Two trials with sodium-glucose cotransporter-2 (SGLT-2) inhibitors showed surprising and unexpected beneficial effects. In fact, after the neutral DPP-4 inhibitor trials the results of the SGLT-2 inhibitor trials took the endocrinology community by surprise. The first trial, the EMPA-REG OUTCOME trial showed in 2015 that the SGLT-2 inhibitor empagliflozin reduced cardiovascular risk and had important additional benefits in terms of reducing heart failure and slowing progression of kidney function decline [18••, 19••]. These results were recently confirmed in the CANVAS study program with the SGLT-2 inhibitor canagliflozin [20••].

The mechanisms for cardiovascular and kidney protection of SGLT-2 inhibitors in patients with diabetes mellitus are incompletely understood. This review summarizes new insights in the potential protective mechanisms of SGLT-2 inhibitors. In the context of recently started renal outcome trials, we will also review the potential benefits of SGLT-2 inhibitors in patients with non-diabetic kidney disease.

### Metabolic Effects of SGLT-2 Inhibitors

In healthy glucose-tolerant individuals with a glomerular filtration rate of 125 ml/min/1.73m^2^ 180 gram glucose is filtered each day by the kidney. In these healthy conditions, urinary glucose concentration is absent owing to an effective reabsorption system, consisting of two sodium-glucose co-transporters (SGLT): SGLT-1 and SGLT-2 [21]. The SGLT-2 transporter is located on the luminal side of the first segment of the proximal tubule in the kidney and is a high-capacity, low-affinity transporter. It is responsible for the reabsorption of approximately 90% of all filtered glucose. The remaining 10% of the filtered glucose is reabsorbed by the low-capacity high-affinity SGLT-1 transporter which is located in more distal segments of the proximal tubule [22]. Both transporters are also located in other organs than the kidney. For example, SGLT-1 transporters actively transport glucose from the lumen into the enterocyte of the small intestine. SGLT-2 transporters are also located in muscles, the heart, brain, and liver [23]. However, it appears that SGLT-2 inhibitors specifically inhibit the SGLT-2 transporter in the proximal tubule of the kidney, demonstrated by a recent study using positron emission tomography with 4-[^18^F]fluoro-dapagliflozin [24]. In patients with diabetes in whom plasma glucose levels exceed 400 mg glucose per 100 ml plasma, SGLT-2 transporters become saturated and the maximum capacity threshold to reabsorb glucose is reached resulting in increased glycosuria [22].

The increased knowledge on the role of the kidney in maintaining glucose homeostasis, in particular the SGLT transporter system, led to the development of drugs inhibiting SGLT. Early experimental studies with phlorizin showed that SGLT inhibition augmented glycosuria and decreased plasma glucose levels [25–27]. However, the clinical development program of this drug was stopped due to gastro-intestinal side effects, which appeared to be caused by blocking SGLT-1 in the gastro-intestinal tract. More selective SGLT-2 inhibitors were subsequently synthesized and investigated. Three of them, dapagliflozin, canagliflozin, and empagliflozin are registered for clinical use in the USA and Europe. Ipragliflozin, tofugliflozin, and luseogliflozin are available in Japan and ertugliflozin and sotugliflozin, combined SGLT-1/SGLT-2 inhibitors, are under investigation. Table 1 shows the molecular structure and the main pharmacokinetic parameters of these SGLT-2 inhibitors.

**Table 1:**
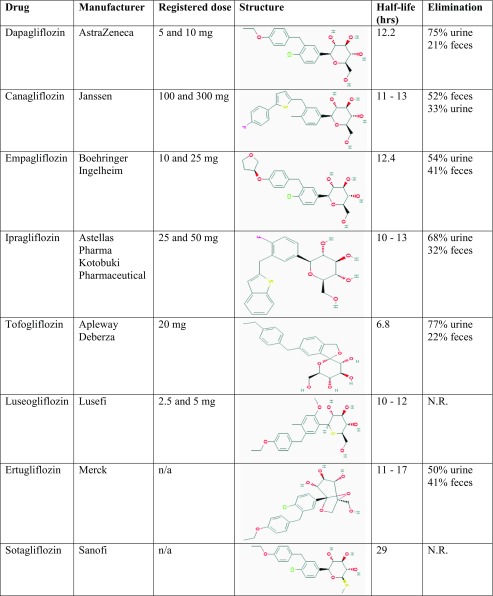
Overview of SGLT-2 inhibitors currently registered or in late phase clinical development

Abbreviations: N.R. not reported

SGLT-2 inhibitors increase urinary glucose excretion by approximately 70–80 gram per day and decrease HbA1c by approximately 0.5 to 0.8% [28]. SGLT-2 inhibitors can be used in combination with other glucose-lowering drugs, and their efficacy to lower HbA1c is not altered when used as adjunct to metformin, sulphonyl urea (SU) derivatives, DDP-4 inhibitors, GLP-1 RA or insulin.

When compared with other glucose-lowering drugs, the efficacy of SGLT-2 inhibitors to lower HbA1c seems modest. However, head-to-head comparisons between SGLT-2 inhibitors and SU derivatives or DPP-4 inhibitors have shown similar efficacy [29–31], and compared with SU derivatives, SGLT-2 inhibitors are more durable over time [31]. The glycemic efficacy appears to depend on the starting HbA1c values with less efficacious effects being observed in patients with low HbA1c values [32, 33]. The lower efficacy at low plasma glucose levels also accounts for the low risk of hypoglycemia observed with this drug class. Another possibility for the low risk of hypoglycemia with SGLT-2 inhibitors is that these agents may stimulate endogenous glucose production [34]. In addition, it is important to note that only approximately 50% of the total filtered glucose is blocked by SGLT-2 inhibitors [35]. This probably contributes to the low risk of hypoglycemia.

SGLT-2 inhibitors are also associated with a consistent 2 to 3 kg weight loss. The effect on body weight, reflecting net calorie loss due to increased glucose excretion, is observed directly after treatment initiation and plateaus after approximately 6 months [36, 37]. The stabilization of body weight loss is likely explained by increased food intake resulting in a new caloric balance. In contrast with other glucose-lowering drugs, weight loss with SGLT-2 inhibitors can be attributed to reductions in both visceral and subcutaneous adipose tissue whereas other glucose-lowering drugs have mainly shown to ameliorate subcutaneous adipose tissue [37]. The clinical relevance of this finding is underscored by studies demonstrating an association of high visceral, but not subcutaneous fat, with increased risk of adverse cardiovascular outcomes [38].

### Natriuresis and Blood Pressure Effects of SGLT-2 Inhibitors

**Fig. 1:**
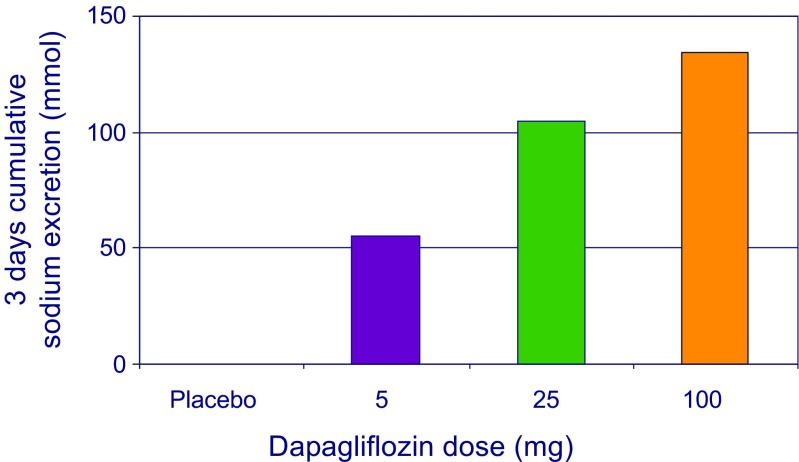
Dapagliflozin induces approximately 55, 105, and 134 mEq negative sodium balance over 3 days at 5, 25, and 100 mg per day respectively. Data derived from reference [39]

The SGLT-2 transporter is responsible for the reabsorption of both glucose and sodium. In addition to glycosuric effects, inhibition of the SGLT-2 transporter also leads to inhibition of proximal sodium reabsorption. Accordingly, in patients with type 2 diabetes who were managed in a carefully controlled environment, it has been shown that dapagliflozin at doses of 5, 25, and 100 mg cause a dose-dependent increase in 3-day sodium excretion ranging from 55 to 134 mmol after 24 h (Fig. 1) [39, 40]. Further research with consecutive 24-h urine sampling over multiple days is ongoing to characterize the magnitude and durability of the natriuretic/diuretic effects of SGLT-2 inhibitors in more detail (clinical trials identifier NCT03152084). The reduction in plasma volume of about 7%, as observed in one study with dapagliflozin in patients with type 2 diabetes and normal renal function, is likely a result of the increased natriuresis and diuresis [41]. In that study, dapagliflozin was directly compared with hydrochlorothiazide. Interestingly, the decrease in body weight during the first 2 weeks with dapagliflozin and hydrochlorothiazide was exactly similar, suggesting that the fall in body weight is not only explained by a net loss of calorie intake but also occurs on the basis of a natriuretic effect. As a consequence of this natriuresis and concurrent osmotic diuresis, blood pressure decreases in response to SGLT-2 inhibition. Indeed, reductions in systolic blood pressure of about 2 to 4 mmHg have been reported in almost all clinical trials with SGLT-2 inhibitors [42]. Based on epidemiologic studies, this would translate into approximately 14% cardiovascular and 18% renal risk reduction [43, 44]. Blood pressure reductions appear to occur both in day time and night time, although not all studies unequivocally demonstrated this [41, 45]. Interestingly, SGLT-2 inhibitors also appear to improve the ability of having a nocturnal fall in blood pressure (dipping) in non-dipping patients with type 2 diabetes. This is of relevance as blood pressure non-dipping patients are at higher risk of cardiovascular events [46]. It should be noted that the blood pressure effects of SGLT-2 inhibitors appear independent of concomitant blood pressure lowering medication. While some have suggested that concomitant renin-angiotensin-aldosterone-system inhibitor or diuretic use may enhance or blunt efficacy of SGLT-2 inhibitors, clinical studies suggest this not to be true [47].

Recently, various studies have documented that the skin can buffer large amounts of sodium independently of extracellular water, body weight, and 24-h urinary sodium excretion. New techniques using ^23^Na-magnetic resonance imaging have become available to measure skin sodium, and it has been shown that high tissue sodium content is associated with cardiovascular risk markers [48]. Six weeks treatment with dapagliflozin has been suggested to reduce skin sodium content which may represent another sodium dependent mechanism by which SGLT-2 inhibitors protect against cardiovascular and heart failure risk [49].

The diuretic effects of SGLT-2 inhibitors are also thought to be involved in improvements in arterial compliance. Optimal blood pressure control is difficult to achieve in patients with DKD and is a key factor contributing to vascular rigidity and stiffness. Aortic pulse wave velocity, an established method to determine arterial stiffness, has been shown to decrease significantly in response to empagliflozin treatment [50]. The reduction in arterial stiffness observed with empagliflozin treatment could not be explained by changes in nitric oxide, renin-angiotensin-aldosterone system activity or sympathetic nervous system activity. Supposedly, other factors mediate the beneficial effects on arterial stiffness such as reduction in weight loss or induction of a negative sodium balance which cause relaxation of vascular smooth muscle cells.

### Cardiovascular Outcome Trials

The cardiovascular protective effects of the SGLT-2 inhibitors empagliflozin and canagliflozin are now established in the EMPA-REG OUTCOME and CANVAS trials (Table 2) [18••, 20••]. The EMPA-REG trial enrolled 7020 participants with type 2 diabetes and established cardiovascular disease who were randomly assigned to empagliflozin 10 or 25 mg or matched placebo. The trial showed after 3.1 years follow-up that empagliflozin resulted in a 14% relative cardiovascular risk reduction (HR, 0.86; 95% confidence interval (CI), 0.74 to 0.99; *p* < 0.001 for non-inferiority, and *p* = 0.04 for superiority) [18••]. Interestingly, a subsequent prespecified analysis showed that empagliflozin had a marked kidney protective effect. The established kidney endpoint of doubling of serum creatinine and initiation of renal replacement therapy occurred less frequently in patients treated with empagliflozin vs. placebo (44 and 55% relative risk reductions, respectively; *p* < 0.001), although the number of these events was small [19••].

**Table 2:** Summary table CANVAS and EMPAREG trials

Study	Population	CV endpoints	CV risk reduction (%)	Renal endpoints	Renal risk reduction (%)	Adverse events
Empagliflozin EMPA-REG (*n* = 7020) [18••, 19••]	Participants with type 2 diabetes, an eGFR > 30 ml/min/1.73m^2^, who were at high risk for cardiovascular (CV) disease	Composite of death from CV causes, non-fatal myocardial infarction, non-fatal stroke	14	Incident/worsening nephropathy	39	Genital infection, urosepsis
				Progression to macroalbuminuria	38	
		Hospitalization for heart failure	35	Doubling serum creatinine	44	
		CV death	38	Renal replacement therapy (RRT)	55	
Canaglifozin CANVAS (*n* = 10,142) [20••]	Participants with type 2 diabetes and an eGFR > 30 ml/min/1.73m^2^, who were ≥ 30 years of age and had a history of symptomatic artherosclerotic CV disease or were ≥ 50 years of age with 2 risk factors for CV disease	Composite of death from CV causes, non-fatal myocardial infarction, non-fatal stroke	14	Progression of albuminuria	27	Fracture risk, amputation, genital infection, volume depletion
				40% reduction in eGFR, RRT, or renal death	40	
		Hospitalization for heart failure	33			
		CV death	13			
		Death from any cause	13			

The results from the CANVAS program were recently reported. The CANVAS program consisted of two randomized controlled trials involving 10,142 participants with either established cardiovascular disease or at risk of cardiovascular disease. Participants were followed for a median of 2.4 years. Similar to the EMPA-REG trial, in the CANVAS program, canagliflozin reduced the relative risks of the primary cardiovascular endpoint by 14% (HR, 0.86; 95% CI, 0.75 to 0.97; *p* < 0.001 for non-inferiority, and *p* = 0.02 for superiority). In addition, canagliflozin also caused a marked 40% reduction in the risks of the primary renal outcome consisting of a 40% estimated glomerular filtration rate (eGFR) decline, the need for renal replacement therapy, and renal death (HR, 0.60; 95% CI, 0.47 to 0.77) [20••].

Despite the impressive results on kidney function, it should be mentioned that the EMPA-REG and CANVAS trials were not designed to assess effects of SGLT-2 inhibitors on the kidney. Therefore, the results should be considered hypothesis generating in terms of kidney outcomes and future prospective trials, as described below, are needed to confirm these findings. Nevertheless, the consistent and impressive results highlight the potential of SGLT-2 inhibitors for the treatment of DKD.

### Renal Protective Pathways

**Fig. 2:**
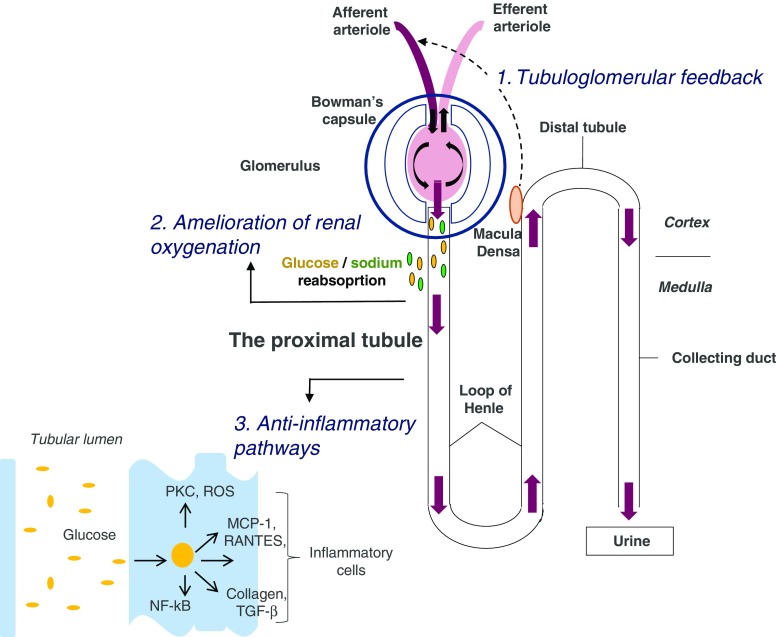
Proposed pathways of renal protective effects: firstly, SGLT2-inhibitors may reduce glomerular hyperfiltration through restoration of tubuloglomerular feedback, an effect which is mediated by increased sodium delivery to the distal tubule. Secondly, SGLT2-inhibitiors ameliorate renal oxygenation owing to reduced tubular workload. Thirdly, SGLT-2 inhibitors may exert anti-inflammatory and anti-fibrotic effects

The protective effects of SGLT-2 inhibitors on the kidney are likely explained by multiple mechanisms. In addition to reductions in HbA1c, blood pressure and body weight, other mechanisms likely contribute to the marked protective effects of SGLT-2 inhibitors on the kidney. These mechanisms include restoration of glomerular feedback, improving renal proximal tubule oxygenation, and suppressing anti-inflammatory and anti-fibrotic pathways (Fig. 2).

Glomerular hyperfiltration is one of the earliest clinical manifestations of DKD and is caused by a complex interplay of diabetes-induced hormonal and structural changes in the nephron. Previous studies, particular in patients with type 1 diabetes, have shown that glomerular hyperfiltration is associated with a higher risk of microalbuminuria and progressive kidney function decline [51]. It is assumed that decreased sodium delivery to the macula densa leads to suppression of tubuloglomerular feedback resulting in afferent vasodilation, increased renal blood flow, and hyperfiltration. SGLT-2 inhibitors increase distal sodium delivery and thereby restore tubuloglomerular feedback and hyperfiltration [52]. This is clinically manifested by an acute drop in eGFR of approximately 4 to 6 ml/min/1.73m^2^ which is completely reversible after SGLT-2 inhibitor cessation, even after years of treatment. Thus, the acute fall in eGFR following SGLT-2 inhibition can in most circumstances be interpreted as a sign of efficacy rather than an adverse effect.

Another mechanism that could potentially explain the protective effects of SGLT-2 inhibitors relates to the improvement in renal hypoxia that is typically observed in diabetic kidneys [53, 54]. The proximal tubule is responsible for the reabsorption of large amounts of water, organic solutes, and electrolytes. These processes are oxygen dependent and cause a decrease in oxygen tension in kidney tissues. The reduction in sodium and glucose reabsorption induced by SGLT-2 inhibitors reduces tubular workload and could ameliorate renal oxygenation resulting in improvements in tubular cell structural integrity and possibly function. Indeed, an experimental study reported that acute SGLT inhibition with phlorizin improved renal cortical oxygen tension in diabetic animals, although unfortunately medullary hypoxia increased [55]. Clearly, more research in humans is required to determine the clinical relevance, but given the central role of hypoxia in DKD, this may represent an important pathway by which SGLT-2 inhibitors confer renal protection.

Inflammation, oxidative stress, and fibrosis are involved in the initiation and progression of kidney disease [56, 57]. Experimental studies have linked SGLT-2 inhibitors with reductions in anti-inflammatory, anti-oxidant and anti-fibrotic markers. For example, MCP-1, NF-kB, levels of 8-OHdG, and l-fatty acid-binding protein (markers of oxidative stress and macrophages) decreased in experimental studies after treatment with ipragliflozin and empagliflozin [58–60]. A recent study translated these preclinical findings to the human situation and demonstrated that 6 weeks treatment with the SGLT-2 inhibitor dapagliflozin decreased urinary levels of the inflammatory markers interleukin-6 and monocyte-attractive-protein-1 [61]. At present, several preclinical and clinical studies examine the effects of SGLT-2 inhibitors on biomarkers of inflammation and fibrosis.

SGLT-2 inhibitors have been shown to lower albuminuria dramatically, possibly by a tubuloglomerular feedback-induced reduction in glomerular hypertension. All registered SGLT-2 inhibitors have consistently shown to lower albuminuria by 30 to 40%. This effect appears to be independent of concomitant ACEi or ARB use and could not be explained by concomitant reductions in HbA1c, blood pressure, or body weight [62–64]. It should be noted that almost all studies investigating the albuminuria-lowering effects of SGLT-2 inhibitors were post-hoc analyses and not primarily designed for this purpose. A recently published prospective randomized cross-over study specifically designed to characterize the albuminuria lowering effect of dapagliflozin confirmed that dapagliflozin decreased albuminuria by 40% compared with placebo treatment [65].

### SGLT-2 Inhibitors in Patients with DKD

The registered SGLT-2 inhibitors in the USA and Europe are not recommended for use in patients with DKD, as it has been shown that their effects on glycemic control attenuates at lower eGFR. It has been hypothesized that the lower glycemic efficacy of SGLT-2 inhibitors in patients with DKD is a result of diminished glucose filtration. Interestingly however, pooled analyses from all phase 3 trials with dapagliflozin and empagliflozin have shown that the efficacy of SGLT-2 inhibitors on other cardiovascular and renal risk factors such as blood pressure, body weight, albuminuria, and uric acid is not attenuated in people with DKD [66–68]. The mechanisms as to why SGLT-2 inhibitors retain their efficacy in lowering these risk markers in patients with DKD is incompletely understood, but it is possible that patients with DKD are more sensitive to mild natriuretic/osmotic diuresis. Regardless of the underlying mechanism, the observed improvements in blood pressure, body weight, and albuminuria in patients with DKD suggest that SGLT-2 inhibitors exert cardiovascular and renal protective effects in this population. Indeed, subgroup analyses of the EMPA-REG trial and the CANVAS program have shown that the effects of empagliflozin and canagliflozin on the primary cardiovascular endpoint are similar in patients with baseline eGFR above and below 60 ml/min/1.73m^2^ [18••, 20••]. However, the number of patients with impaired kidney function in these trials was low (e.g., 26% in the EMPA-REG trial), and therefore the number of patients who required dialysis, the clinically meaningful endpoint in trials of kidney disease progression, was less than 30 [19••]. This is no surprise since the trials were not designed to assess effects of empagliflozin and canagliflozin on kidney endpoints. The ongoing CREDENCE and DAPA-CKD trials (clinical trials identifier NCT02065791 and NCT03036150) are specifically designed to establish the safety and efficacy of canagliflozin in slowing kidney progression in patients with DKD. Design and patient characteristics of the CREDENCE and DAPA-CKD trial are described in Table 3.

**Table 3:** Study characteristics of the CREDENCE and DAPA-CKD trials

	CREDENCE	DAPA-CKD
Clincial trials identifier	NCT02065791	NCT03036150
Design	Randomized placebo-controlled double-blind trial	Randomized placebo-controlled double-blind trial
Study Population	Type 2 diabetes	Chronic kidney disease
	UACR 300–3500 mg/g	UACR 200–3500 mg/g
	eGFR 30–90 ml/min/1.73m^2^	eGFR 30–75 ml/min/1.73m^2^
Primary endpoint	Composite of end-stage renal disease, doubling of serum creatinine, renal or cardiovascular death	Composite of end-stage renal disease, ≥ 50% eGFR decline, renal or cardiovascular death
Secondary endpoints	-Cardiovascular death -All-cause death -Cardiovascular composite endpoint (myocardial infarction, stroke, CV death) -Renal composite endpoint (end-stage renal disease, doubling of serum creatinine, renal death)	-All-cause death First occurrence of: -End-stage renal disease, ≥ 50% eGFR decline, renal death -Cardiovascular death or hospitalization for heart failure
Number of patients	4464	4000
Anticipated trial completion	June 2019	November 2020

### SGLT-2 Inhibitors in Non-diabetic Kidney Disease

**Fig. 3:**
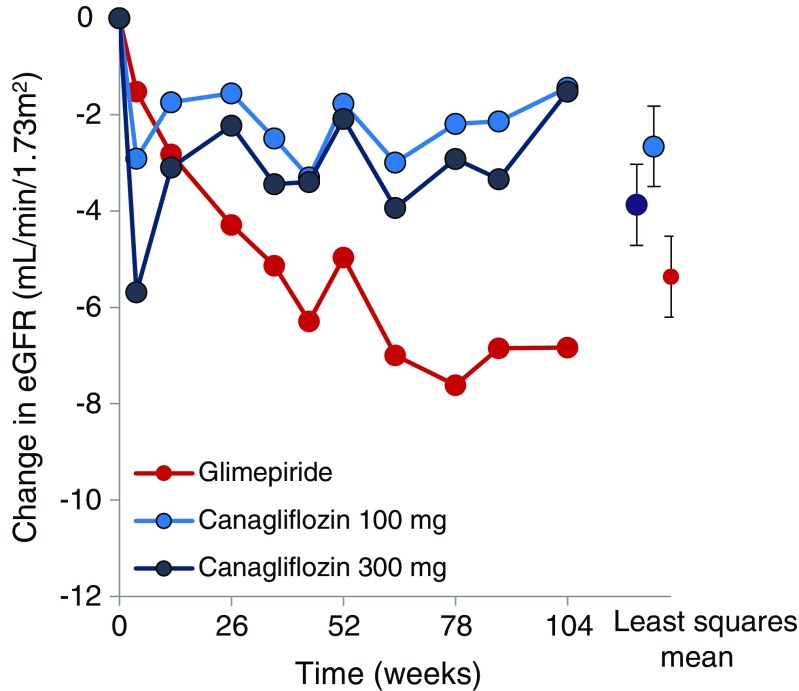
Canagliflozin induces an acute fall in eGFR during the first 4 weeks of treatment followed by a stabilization of eGFR decline during subsequent 2 years follow-up, whereas eGFR progressively declined over time during treatment with glimepiride. HbA1c levels were similar at baseline and during follow-up in the canagliflozin and glimepiride group. (Republished with permission from the American Society of Nephrology from Heerspink et al. [69])

The EMPA-REG trial and CANVAS trials showed that the Kaplan-Meier curves for the renal endpoint diverged already during the initial months of the trial. Remarkably, a 2-year clinical trial comparing head-to-head canagliflozin with glimepiride showed that with canagliflozin the rate of kidney function decline was significantly lower while glycemic control was similar between the two classes (Fig. 3) [69]. These data combined indicate that the protective effects of SGLT-2 inhibitors are unlikely to be mediated by improvements in glycemic control. The restoration of tubuloglomerular feedback along with glomerular afferent vasoconstriction is thought to be an important mechanism accounting for the protective effects of SGLT-2 inhibitors on kidney function. Based on these non-glycemic effects, there is a strong imperative to extend the use of SGLT-2 inhibitors to non-diabetic chronic kidney diseases (CKD) that are also characterized by glomerular hypertension, hyperfiltration, and significant albuminuria. For example, various studies have documented that obesity induced CKD is associated with altered kidney hemodynamics leading to increased renal plasma flow and GFR (i.e., hyperfiltration) [70]. The mechanism is likely a result of increased reabsorption of sodium in the proximal tubule leading to altered tubule-glomerular feedback. In hypertensive nephrosclerosis, loss of autoregulation of the afferent and efferent arteriole in the kidney drive a hyperfiltration type of glomerular lesion and subsequently kidney damage [71]. Immunoglobulin A (IgA) nephropathy is another CKD subtype which could benefit from SGLT-2 inhibitors. Patients often present with significant proteinuria which is a strong determinant of renal prognosis. Current therapy for these patients consists of RAAS inhibition, as well as immunosuppression, but is insufficient in a considerable proportion of patients [72–74]. SGLT-2 inhibition may be a promising adjunctive therapy to further lower intra-glomerular pressure and proteinuria. Secondary focal segmental glomerulo-sclerosis (FSGS) is characterized by hypertension, significant proteinuria, and is caused by a reduction in renal mass due to various causes such as surgical ablation, sickle cell anemia, or episodes of active glomerulonephritis [75]. Importantly, hyperfiltration is common in all the aforementioned disorders. Reversing the hyperfiltering state through pharmacological interventions, such as with SGLT-2 inhibitors, may improve the renal outcome of these patients [76].

An important question to be addressed is of course whether it is safe to use SGLT-2 inhibitors in non-diabetic CKD patients. As described above, the risk of hypoglycemia with SGLT-2 inhibitors in non-diabetic patients is likely low as the degree of glucose-lowering positively correlates with the filtered glucose load which is low in non-diabetic CKD populations. Clinical experience with SGLT-2 inhibitors in non-diabetic conditions is currently limited, but studies in healthy volunteers have shown that SGLT-2 inhibitors can be safely used at high doses, even up to 50-fold the dose used in clinical practice, without inducing hypoglycemia [77]. Thus, the currently available data suggest that SGLT-2 inhibitors can be safely used in non-diabetic conditions at least with respect to hypoglycemia. A number of trials, such as the DIAMOND (NCT03190694) and DAPA-CKD (NCT03036150), are currently ongoing to investigate the efficacy and safety of SGLT-2 inhibitors in patients with non-diabetic CKD. These trials are due to report in 2019 and 2021.

### Who Should Be Treated with SGLT-2 Inhibitors?

Current guidelines recommend to use SGLT-2 inhibitors as adjunct to metformin therapy in patients with type 2 diabetes and established cardiovascular disease [78]. This recommendation is based on the EMPA-REG trial. A well-known side effect of SGLT-2 inhibitors is genital infections, probably mediated by glycosuria. Patients with multiple episodes of genital infections are therefore not the ideal candidates to start an SGLT-2 inhibitor. Physicians are also advised to be careful with SGLT-2 prescription in patients at risk of diabetic ketoacidosis (DKA), as this may be an important although rare serious adverse event related to this drug class. Of note, the risk of DKA with SGLT-2 inhibitors is low in patients with type 2 diabetes, which may explain why DKA signals were not detected in clinical trials. However, based on administrative databases and real-world evidence a 2-fold increased risk of DKA has been suggested when compared with DPP-4 inhibitors [79]. It should be noted however that other database studies suggested no increased risk of DKA with SGLT-2 inhibitors [80].

Volume depletion is another adverse effect detected in clinical trials. Patients at risk of volume depletion, such as those with gastrointestinal fluid losses or reduced oral intake, are therefore not good candidates to start SGLT-2 inhibitors. In addition, SGLT-2 inhibitors should be withheld during procedures that may reduce renal perfusion such as elective surgery or intravenous contrast procedures in the same way as is done with ACE inhibitors and angiotensin receptor blockers. Along the same line, SGLT-2 inhibitors should not be continued during short-term periods of NSAID use, as the combination of these two drug classes may reduce renal perfusion and may evoke acute kidney injury.

The CANVAS program unexpectedly showed that patients treated with canagliflozin had a 2-fold higher risk of lower limb amputations compared with placebo-treated patients. Patients with a history of amputations or with peripheral vascular disease were at highest absolute risk for amputations during the trial, but the relative effect of canagliflozin was similar across subgroups [20••]. The underlying mechanism of this potential adverse event is unclear nor is it known whether this represents a class effect or an effect particularly associated with canagliflozin. A recent analysis of the FDA pharmacovigilance database suggested that canagliflozin use, but not dapagliflozin or empagliflozin use, may be associated with an increased risk of amputations [81]. However, causality cannot be assessed based on pharmacovigilance databases and future trials in high-risk populations are needed to determine whether peripheral ischemia necessitating amputations are a serious adverse event of this drug. This adverse event complicates the management of patients with peripheral vascular disease. On the one hand, they had the highest risk of amputations in CANVAS, but they also experienced a marked benefit with respect to cardiovascular outcomes. Until other data becomes available, it seems reasonable to treat these individuals with dapagliflozin or empagliflozin, since to date no safety signal has emerged with these two SGLT-2 inhibitors.

## Conclusion

SGLT-2 inhibitors are promising. Not only do they improve glycemic control but they also offer substantial protection against progression of cardiovascular and kidney disease in patients with diabetes independent of glucose control. In the last 2 years, several hypotheses emerged that could explain the underlying mechanisms of these protective effects. With respect to the kidney, restoring tubuloglomerular feedback, and attenuating intra-renal hypoxia and inflammation seem plausible mechanisms by which SGLT-2 inhibitors delay the rate of kidney function decline. These glucose-independent effects offer the opportunity to reposition SGLT-2 inhibitors to the non-diabetic CKD population. Ongoing trials will determine whether this novel drug class is indeed an important asset to combat the epidemic of diabetic as well as non-diabetic CKD.
